# Supplementary figures and images for: Screening macrophage polarization genes in spinal cord injury as therapeutic targets
Source: PLoS One. 2026 May 4;21(5):e0347599. doi: 10.1371/journal.pone.0347599 (PMC13138653; doi:10.1371/journal.pone.0347599)

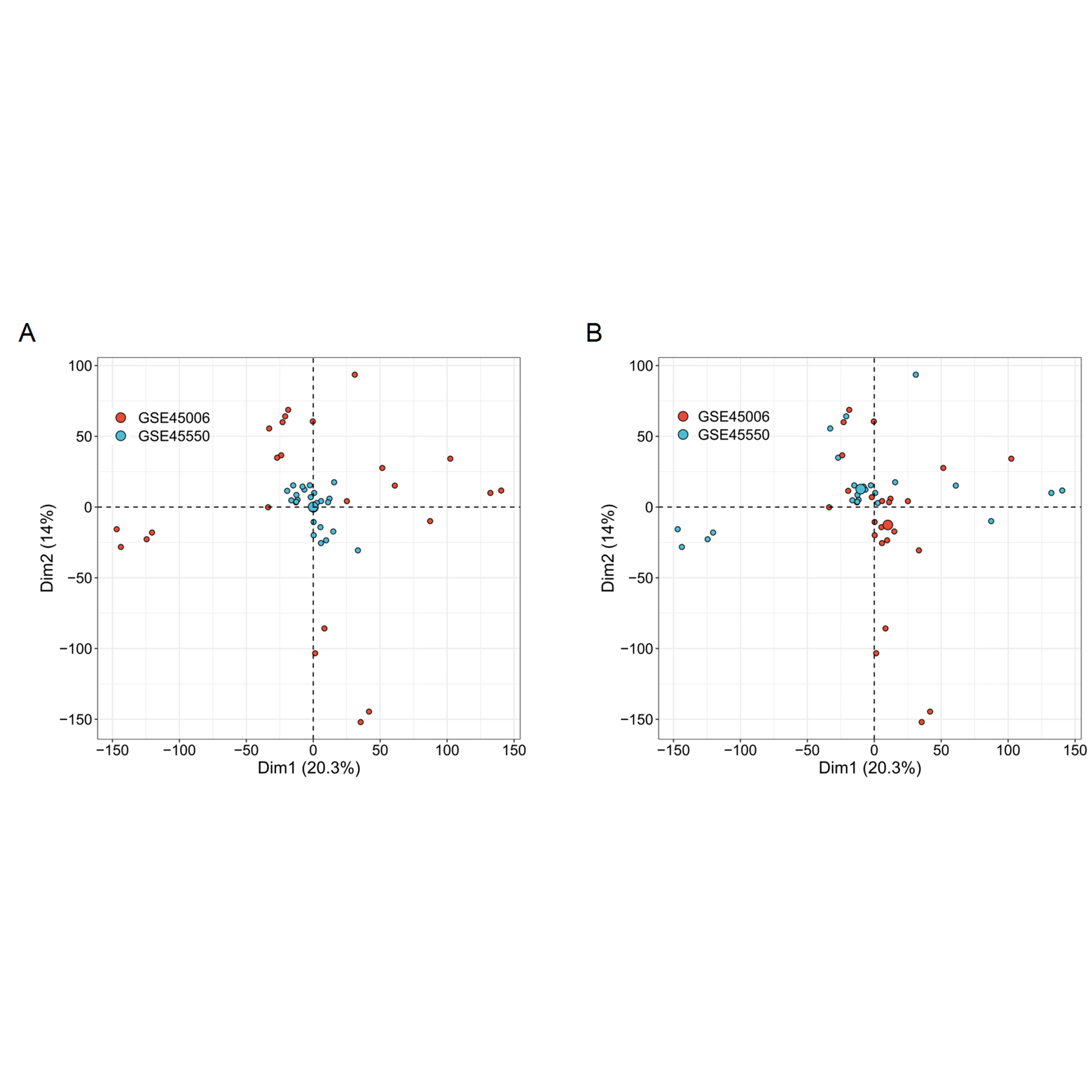

Supplement: S1 Fig — (A) Before batch effect correction. (B) After batch effect correction, showing excellent batch integration. (TIF) [file pone.0347599.s007.TIF]

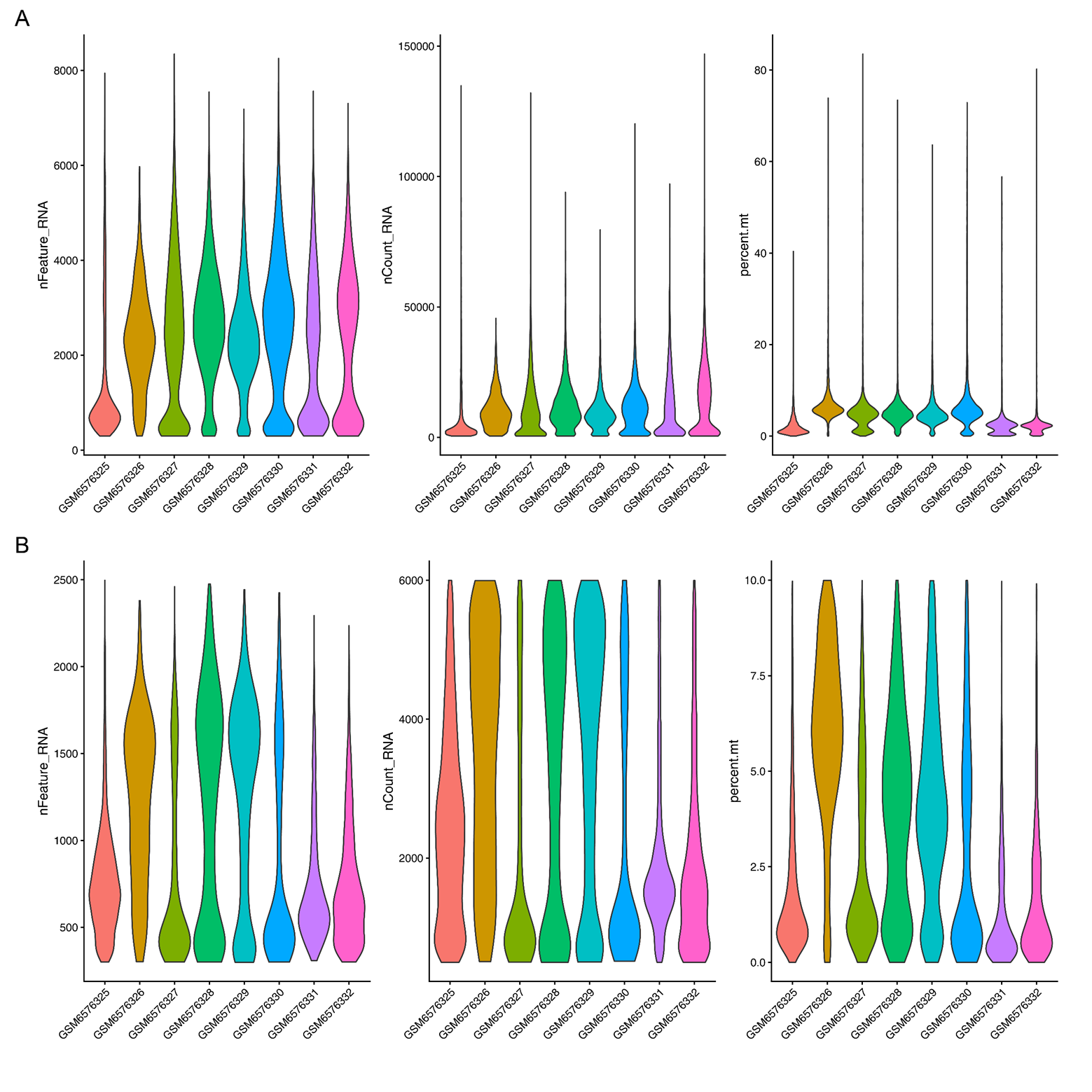

Supplement: S2 Fig — (A, B) Distribution plots of nFeature_RNA, nCount_RNA, and percent.mt before (A) and after quality control (B). (TIF) [file pone.0347599.s008.TIF]
