# Supplementary material for: Screening macrophage polarization genes in spinal cord injury as therapeutic targets
Source: PLoS One. 2026 May 4;21(5):e0347599. doi: 10.1371/journal.pone.0347599 (PMC13138653; doi:10.1371/journal.pone.0347599)
Supplement: Attachment — Submitted filename: PONE-D-25-22705-reporting.pdf [file pone.0347599.s012.pdf]

REVIEWER'S REPORT TO MANUSCRIPT ID (PLOS ONE): PONE-D-25-22705

Decision: Minor revision

Title: **Screening macrophage polarization genes in spinal cord injury as therapeutic targets**

27.05.2025

INTRODUCTORY COMMENT

In the manuscript titled *"Screening macrophage polarization genes in spinal cord injury as therapeutic targets"*, Xiaowei et al. attempt to explore an understudied area of macrophage polarization in spinal cord injury (SCI). To achieve this, the authors adopted a fairly comprehensive bioinformatics analysis, ranging from screening differential gene expression analysis (MPRGs) to miRNA/TF regulatory network construction. Furthermore, the authors explored external validation of the three final DEGs (via qPCR testing). Overall, the study is sound, timely, and worth considering. However, the manuscript can further be fine-tuned by addressing a couple of sections.

CORE STRENGTHS

- *Standard bioinformatics pipeline:* Robust feature-selection techniques (random forest, LASSO, and gradient boosting) and enrichment methods were used to screen the differentially expressed genes. Performing batch correction via the sva package is particularly commendable.

- *Immunoinfiltration and function pathway analysis:* The authors used ssGSEA to highlight functional pathway enrichment of the screened genes, as well as using CIBERSORT to underscore the role of the identified genes in the immune cell infiltration within the tumor microenvironment.

- *Regulatory and gene-drug interaction network:* The authors further performed TF-miRNA and drug-gene interaction analysis to illustrate or reveal translational relevance.

- *External validation:* Though not a strong point, performing *in vivo* qPCR validation in rat models adds further insights (with respect to predictions).

WEAKNESSES

- *Pre-processing:* Though it is mentioned that batch correction was performed (i.e., using the sva package), the manuscript is quite silent on key data pre-processing methods. This aspect should be addressed. The datasets used are described in moderate detail (e.g., platform types like GPL1355 and GPL4135). Additionally, the sample sizes are small, which implies the authors ought to describe how they dealt with sample-size bias.

- *Lack of in-depth discussion of the SCI hub genes:* A detailed contextual discussion of the screened genes in SCI would further strengthen the manuscript. While gene ontology (GO/KEGG) was performed, discussion of the MPGRs with respect to past studies has not been thoroughly discussed.

- *Language clarity:* The language can further be improved. There are some weird spelling errors (aftereffects → after-effects). Italicize the 'genus' and 'species' names (e.g., Rattus norvegicus → *Rattus norvegicus*). Using the word *'gained'* in the expression *"Firstly, the DE-MPRGs were gained through taking the intersection"* does not quite appear standard.

- *Limitations of the study:* Clearly, this study has a couple of limitations not mentioned in the manuscript. For example, single-cell RNA analysis (Seurat package) was performed. Therefore, the authors could dedicate a paragraph to pointing out areas lacking in the current study, as well as further direction (if any).
