# Supplementary material for: Screening macrophage polarization genes in spinal cord injury as therapeutic targets
Source: PLoS One. 2026 May 4;21(5):e0347599. doi: 10.1371/journal.pone.0347599 (PMC13138653; doi:10.1371/journal.pone.0347599)
Supplement: Attachment — Submitted filename: Report-manuscript-PONE-D-25-22705R1.pdf [file pone.0347599.s014.pdf]

PLOS One

Manuscript Number: PONE-D-25-22705R1

This revised manuscript has been significantly improved. In the original version, my concerns largely revolved around issues of preprocessing (e.g., small sample size), lack of detailed discussion of the SCI hub genes screened, language clarity, and failure to acknowledge the limitations of the present study. All these issues have largely been addressed now.

Minor suggestion:

Since the GSE45006 and GSE45550 datasets were used to screen the DEGs (using the `limma` package) for the downstream analyses, the findings in the study can further be strengthened using the DExMA package or the `Robust Rank Aggregation` (RRA) package for meta-analysis of the GSE45006 and GSE45550 datasets. Using these two packages, can the approach used in DOI: https://doi.org/10.3390/diagnostics15141770 be applicable to your study? How are your findings strengthened?

Since links to web tools are susceptible to changes in the future, the authors are strongly advised to include the date accessed for the web links in their study. As an example, the authors state

"Queries in the Drug-Gene Interaction Database (DGIdb; https://dgidb.genome.wustl.edu/) relied on individual hub genes …"

It should be

"Queries in the Drug-Gene Interaction Database (DGIdb; https://dgidb.genome.wustl.edu/ [Accessed: June 23, 2025]) relied on individual hub genes …" Notice that in the second case, we indicate the web tool was accessed on June 23, 2025.
