# Supplementary material for: Screening macrophage polarization genes in spinal cord injury as therapeutic targets
Source: PLoS One. 2026 May 4;21(5):e0347599. doi: 10.1371/journal.pone.0347599 (PMC13138653; doi:10.1371/journal.pone.0347599)
Supplement: Attachment — Submitted filename: Response to Reviewers.docx [file pone.0347599.s015.docx]

**Point-by-point response to the editor and reviewer comments**

**Dear Editor and Reviewers,**

Thank you very much for your thoughtful comments and valuable suggestions, which have greatly improved the quality of our manuscript. We sincerely appreciate your efforts and guidance throughout the review process.

We have carefully revised the manuscript in accordance with all the comments provided. A detailed, point-by-point response to each comment is attached below. We have made every effort to address all concerns, and we hope that the revised version will now meet the standards for publication in *PLOS ONE*.

Please feel free to contact us if any further questions arise. We would be glad to provide additional clarification.

**Journal Requirements:**

When submitting your revision, we need you to address these additional requirements.

1. Please ensure that your manuscript meets PLOS ONE's style requirements, including those for file naming.

Re: We sincerely thank the *PLOS ONE* editorial team for reviewing our manuscript and providing valuable comments. We have carefully considered all suggestions and have thoroughly revised the manuscript to comply with the formatting requirements and editorial guidance provided.

2. Please include a complete copy of PLOS’ questionnaire on inclusivity in global research in your revised manuscript. Our policy for research in this area aims to improve transparency in the reporting of research performed outside of researchers’ own country or community. The policy applies to researchers who have travelled to a different country to conduct research, research with Indigenous populations or their lands, and research on cultural artefacts. The questionnaire can also be requested at the journal’s discretion for any other submissions, even if these conditions are not met. Please find more information on the policy and a link to download a blank copy of the questionnaire here: https://journals.plos.org/plosone/s/best-practices-in-research-reporting. Please upload a completed version of your questionnaire as Supporting Information when you resubmit your manuscript.

Re: We have carefully reviewed PLOS’ policy on inclusivity in global research and completed the corresponding questionnaire as required, and appropriately cited it in the Materials and methods section (Line 280-282). The completed questionnaire has been included as a Supporting Information file with the revised manuscript, and we believe this addition further enhances the transparency of our study.

3. We noticed you have some minor occurrence of overlapping text with the following previous publication(s), which needs to be addressed:

https://www.sciencedirect.com/science/article/pii/S2950588725000424?via%3Dihub

In your revision ensure you cite all your sources (including your own works), and quote or rephrase any duplicated text outside the methods section. Further consideration is dependent on these concerns being addressed.

Re: We sincerely thank the editor for the careful review of our manuscript and for providing valuable comments regarding textual presentation. We have taken the issue of textual overlap very seriously and conducted a thorough check and revision of the entire manuscript. During the revision process, we carefully cross-checked all cited references and substantively rewrote and restructured sections of the Introduction, Discussion, and other parts where overlaps were identified, ensuring originality in language expression. While some bioinformatic analysis methods, such as hub gene selection via machine learning, are standard in the field, the novelty of this study lies in the integration of transcriptomic data with single-cell RNA-seq, allowing higher-resolution analysis of macrophage subpopulation dynamics, and in the experimental validation of selected hub genes in vivo—work that has not been systematically reported in the existing literature. We have conducted a similarity/plagiarism check on the manuscript and confirm that the revised version fully addresses any issues of textual overlap, ensuring original language and proper citation throughout. We appreciate your guidance, which has helped us more clearly highlight the unique contributions of this study, and we respectfully submit the manuscript for further consideration.

4. Thank you for stating the following financial disclosure:

“This work was supported by General Programs of Natural Science Research of Anhui Provincial Education Department (Grant No. ZR2022B001).”

Please state what role the funders took in the study. If the funders had no role, please state: "The funders had no role in study design, data collection and analysis, decision to publish, or preparation of the manuscript."

If this statement is not correct you must amend it as needed.

Please include this amended Role of Funder statement in your cover letter; we will change the online submission form on your behalf.

Re: Thank you very much for reviewing our manuscript and providing valuable comments. In response to the request for an updated financial disclosure statement, we have complied accordingly. The funder (General Programs of Natural Science Research of Anhui Provincial Education Department, Grant No. ZR2022B001) had no role in any aspect of this study. Accordingly, we have included the following revised statement in our cover letter:

"This work was supported by the General Programs of Natural Science Research of Anhui Provincial Education Department (Grant No. ZR2022B001). The funders had no role in study design, data collection and analysis, decision to publish, or preparation of the manuscript."

We confirm that this statement is accurate. We would appreciate the journal’s assistance in updating the corresponding information in the online submission system. Thank you again for your guidance.

5. If the reviewer comments include a recommendation to cite specific previously published works, please review and evaluate these publications to determine whether they are relevant and should be cited. There is no requirement to cite these works unless the editor has indicated otherwise.

Re: We sincerely thank the editor for reviewing our manuscript and for providing valuable comments. We have taken note of the suggestion regarding reference citations. We carefully reviewed and evaluated the publications mentioned by the reviewers and have cited them judiciously in the revised manuscript where appropriate.

**Reviewers' comments:**

Reviewer's Responses to Questions

Comments to the Author

1. Is the manuscript technically sound, and do the data support the conclusions?

The manuscript must describe a technically sound piece of scientific research with data that supports the conclusions. Experiments must have been conducted rigorously, with appropriate controls, replication, and sample sizes. The conclusions must be drawn appropriately based on the data presented.

Reviewer #1: Yes

Reviewer #2: Yes

Re: We sincerely thank you for your recognition of the technical rigor, data reliability, and validity of the conclusions in our study. We are pleased that the scientific quality of our manuscript has been acknowledged. We remain committed to rigorous research and have carefully revised and improved the manuscript based on the other suggestions provided.

2. Has the statistical analysis been performed appropriately and rigorously?

Reviewer #1: Yes

Reviewer #2: Yes

Re: We sincerely thank you for acknowledging the rigor and appropriateness of the statistical analyses in our study. We remain committed to conducting our research with objective and rigorous statistical standards.

3. Have the authors made all data underlying the findings in their manuscript fully available?

The PLOS Data policy requires authors to make all data underlying the findings described in their manuscript fully available without restriction, with rare exception (please refer to the Data Availability Statement in the manuscript PDF file). The data should be provided as part of the manuscript or its supporting information, or deposited to a public repository. For example, in addition to summary statistics, the data points behind means, medians and variance measures should be available. If there are restrictions on publicly sharing data—e.g. participant privacy or use of data from a third party—those must be specified.

Reviewer #1: No

Reviewer #2: No

Re: Thank you for reviewing our manuscript and providing valuable comments. We fully recognize the shortcomings regarding data availability and take this issue seriously. To comply with PLOS data policies, we have submitted the raw data underlying all statistical figures/charts as Supporting Information, and updated the Data Availability Statement to include repository names, accession numbers, and direct access links, ensuring that all data are publicly accessible without restriction. Additionally, key datasets supporting critical results are provided alongside the manuscript to enhance reproducibility and transparency. We are committed to fully implementing these changes in the revised manuscript, ensuring that all data are completely accessible, traceable, and in compliance with journal requirements, and we sincerely appreciate your suggestions for improving the scientific rigor of our work.

4. Is the manuscript presented in an intelligible fashion and written in standard English?

PLOS ONE does not copyedit accepted manuscripts, so the language in submitted articles must be clear, correct, and unambiguous. Any typographical or grammatical errors should be corrected at revision, so please note any specific errors here.

Reviewer #1: No

Reviewer #2: Yes

Re: We sincerely thank the reviewer for the valuable comments, particularly regarding the clarity and precision of the language. In response to the issues highlighted by Reviewer #1, we have thoroughly polished the manuscript, correcting grammatical and spelling errors, inappropriate expressions, and potentially ambiguous sentences, while also optimizing the logic and structure and reorganizing some lengthy or obscure paragraphs to ensure clear, accurate, and easily understandable presentation. The revised manuscript reflects all these improvements, and we greatly appreciate the meticulous work of the reviewers and editors, which has been instrumental in enhancing the quality of our study.

5. Review Comments to the Author

Please use the space provided to explain your answers to the questions above. You may also include additional comments for the author, including concerns about dual publication, research ethics, or publication ethics. (Please upload your review as an attachment if it exceeds 20,000 characters)

Reviewer #1: Introductory comment

--------------------------

In the manuscript titled “Screening macrophage polarization genes in spinal cord injury as therapeutic targets”, Xiaowei et al. attempt to explore an understudied area of macrophage polarization in spinal cord injury (SCI). To achieve this, the authors adopted a fairly comprehensive bioinformatics analysis, ranging from screening differential gene expression analysis (MPRGs) to miRNA/TF regulatory network construction. Furthermore, the authors explored external validation of the three final DEGs (via qPCR testing). Overall, the study is sound, timely, and worth considering. However, the manuscript can further be fine-tuned by addressing a couple of sections.

Core strengths

-----------------

Standard bioinformatics pipeline: Robust feature-selection techniques (random forest, LASSO, and gradient boosting) and enrichment methods were used to screen the differentially expressed genes. Performing batch correction via the sva package is particularly commendable.

Immunoinfiltration and function pathway analysis: The authors used ssGSEA to highlight functional pathway enrichment of the screened genes, as well as using CIBERSORT to underscore the role of the identified genes in the immune cell infiltration within the tumor microenvironment.

Regulatory and gene-drug interaction network: The authors further performed TF-miRNA and drug-gene interaction analysis to illustrate or reveal translational relevance.

External validation: Though not a strong point, performing in vivo qPCR validation in rat models adds further insights (with respect to predictions).

Weaknesses

--------------

Pre-processing: Though it is mentioned that batch correction was performed (i.e., using the sva package), the manuscript is quite silent on key data pre-processing methods. This aspect should be addressed. The datasets used are described in moderate detail (e.g., platform types like GPL1355 and GPL4135). Additionally, the sample sizes are small, which implies the authors ought to describe how they dealt with sample-size bias.

Re: We sincerely thank you for your recognition of the value of our study and for your insightful comments regarding data preprocessing and sample size, which have been crucial in enhancing the rigor and clarity of our manuscript. In response, we have expanded the “Materials and Methods” section to provide detailed information on the quality control steps performed prior to batch correction and to explain the statistical rationale for the chosen preprocessing procedures, ensuring data reliability and comparability. We have also addressed concerns regarding the limited sample size by emphasizing in the Methods the use of robust statistical approaches and cross-validation techniques to reduce the risk of overfitting, and by acknowledging in the Discussion this limitation while highlighting the need for validation in larger independent cohorts. These revisions aim to present the study’s limitations more comprehensively and demonstrate our careful consideration of these issues. We greatly appreciate your insightful comments, which have significantly improved the quality of our manuscript, and we hope that these modifications fully address your concerns.

Lack of in-depth discussion of the SCI hub genes: A detailed contextual discussion of the screened genes in SCI would further strengthen the manuscript. While gene ontology (GO/KEGG) was performed, discussion of the MPGRs with respect to past studies has not been thoroughly discussed.

Re: We sincerely thank you for this insightful and constructive comment. We fully agree that a thorough comparison and interpretation of the selected hub genes within the context of existing literature is crucial for enhancing the academic value of the manuscript. Following your suggestion, we have revised and expanded the Discussion section. Rather than merely describing the results of GO/KEGG enrichment analyses, we have examined each of the final hub genes in the context of current literature, deepening the discussion of their known functions in SCI or other neurological disorders and the consistency of our findings with previous studies. We have also integrated insights from the literature and single-cell analyses to elucidate the relationships between these genes and macrophage polarization, highlighting the novelty of our study in identifying potential key targets. We believe that these additions and elaborations now allow the Discussion section to more fully interpret the potential significance of our findings. We greatly appreciate your guidance, which has significantly strengthened the depth of our manuscript.

Language clarity: The language can further be improved. There are some weird spelling errors (aftereffects → after-effects). Italicize the ‘genus’ and ‘species’ names (e.g., Rattus norvegicus → *Rattus norvegicus*). Using the word ‘gained’ in the expression “Firstly, the DE-MPRGs were gained through taking the intersection” does not quite appear standard.

Limitations of the study: Clearly, this study has a couple of limitations not mentioned in the manuscript. For example, single-cell RNA analysis (Seurat package) was performed. Therefore, the authors could dedicate a paragraph to pointing out areas lacking in the current study, as well as further direction (if any).

Re: We sincerely thank you for your specific and valuable suggestions regarding language details and the limitations of our study. We have carefully revised the manuscript based on each of your comments.

1. **Language clarity:** We have thoroughly proofread and polished the entire manuscript. Specific revisions include correcting spelling errors, ensuring proper formatting of all biological genus and species names, and replacing non-standard expressions with more precise academic language.
2. **Study limitations:** We fully agree with your points and have added a dedicated section on limitations and future directions at the end of the Discussion. This section addresses potential biases associated with retrospective analyses based on public databases, the inherent limitations of bulk RNA-seq data in distinguishing cell types, the current shortcomings of scRNA-seq analyses in precisely defining continuous phenotypes, and the preliminary nature of the in vivo validation. Corresponding future research directions are also proposed to enhance the completeness and scientific rigor of the study.

We greatly appreciate your meticulous review, which has substantially improved the quality of our manuscript.

Reviewer #2: The manuscript is comprehensive and methodologically sound, providing a solid foundation for the study’s conclusions. The study offers a comprehensive bioinformatics analysis aimed at identifying macrophage polarization-related genes involved in spinal cord injury (SCI). The multi-step approach —encompassing DEG screening, GO/KEGG enrichment analyses, machine learning algorithms (LASSO, XGBoost, RF), gene-gene interaction networks, and drug prediction — is well-designed and leads to valuable insights. Addressing the following points with additional details and clarifications would improve transparency, and reproducibility.

- It would enhance reproducibility if the authors provide more information on the specific parameters or methods used for batch effect correction. Additionally, including a visualization (e.g., PCA or MDS plots) before and after batch effect removal would help readers assess the effectiveness of this step.

Re: We sincerely thank you for your recognition of our work and for your valuable suggestions to enhance the transparency and reproducibility of the manuscript. In response to your comments, we have made the following additions and revisions. The “Materials and Methods” section now includes detailed descriptions of the batch correction methods used, including specific steps and key parameters, to ensure full transparency of the analytical workflow. To visually demonstrate the effect of batch correction, a figure has been provided as Supporting Information (S1 Fig). We greatly appreciate your insightful comments.

- I recommend using GridSearchCV to improve the performance of each machine learning algorithms.

Re: We sincerely thank you for your valuable suggestion regarding the use of GridSearchCV to optimize model performance. We fully agree that systematic hyperparameter tuning is essential for ensuring optimal performance of machine learning models. We would like to clarify that, although the specific function name “GridSearchCV” was not explicitly mentioned in the Methods, the core concept has been fully implemented in our analysis workflow—that is, hyperparameter optimization through grid search combined with cross-validation. Specifically, the hyperparameter tuning logic was applied during model construction for each algorithm (LASSO, Random Forest, and XGBoost), with detailed search spaces defined for key parameters and performance evaluated via cross-validation. This process is fully consistent with the built-in logic of GridSearchCV. To ensure clarity and reproducibility, we have added the detailed procedures and parameters for all three machine learning algorithms in the Methods section. We believe that the optimization approach we employed is equivalent to GridSearchCV in both purpose and implementation. We greatly appreciate your insightful comments, which have prompted us to present this important methodological detail more clearly.

- The application of the “limma” package with clear thresholds (P < 0.05, |log2FC| > 0.5) is appropriate for identifying DEGs. Functional enrichment via GO and KEGG pathways using “clusterProfiler” and “org.Rn.eg.db” packages is well described. Nevertheless, the authors should clarify whether multiple testing corrections (e.g., FDR adjustment) were applied and specify the exact adjusted P-value thresholds for enrichment analyses.

Re: We sincerely thank you for acknowledging the bioinformatic analysis methods used in our study. Your comment regarding multiple hypothesis testing correction is highly important, as it directly impacts the rigor of result interpretation. In response, we have clarified and expanded the “Materials and Methods” section. During differential expression analysis, the resulting p-values were corrected for false discovery rate (FDR) using the Benjamini-Hochberg method. The threshold of *P* < 0.05 reported in the manuscript refers to the FDR-adjusted p-values (adjusted *P* < 0.05), combined with |log2FC| > 0.5 as the criterion for selecting differentially expressed genes (provided in the supplementary table). For functional enrichment analyses, adjusted *P* < 0.05 was also applied to determine significantly enriched pathways or functional terms. The method for p-value adjustment has been explicitly included in the statistical analysis section. These additions ensure that our statistical workflow fully adheres to current standards for high-throughput data analysis, thereby enhancing the reliability of the results. We greatly appreciate your attention to this critical detail, which has helped make the methodological description more complete and rigorous.

- Employing three machine learning algorithms (LASSO, Random Forest, and XGBoost) for biomarker selection is a strong approach. However, detailed information about the tuning parameters, cross-validation strategy, and feature selection criteria for each algorithm is missing and should be provided to enhance reproducibility. Also, a brief description of how the final biomarker set was validated (e.g., internal validation metrics or external validation) would strengthen this section.

Re: We sincerely thank you for recognizing the integrated machine learning approach used in our study and for your specific suggestions to enhance methodological reproducibility. We fully agree that providing detailed parameters and validation information is essential. In response to your comments, we have expanded and refined the “Materials and Methods” section. Specifically, we have included detailed parameters and strategies for the machine learning algorithms, as well as the integration and validation methods for the final hub genes. We also emphasized the use of external datasets and in vivo experiments to validate the stability and reliability of the expression trends of the hub genes. We believe that these comprehensive additions will greatly facilitate the reproducibility of our analysis workflow and provide a clearer understanding of the complete research logic, from computational prediction to experimental validation. We greatly appreciate your guidance, which has substantially improved the transparency of our methodology.

- Given the study's focus on macrophage polarization, incorporating or referencing single-cell transcriptomics data would improve resolution and specificity. The authors are encouraged to discuss this limitation more explicitly in the discussion section.

Re: We sincerely thank you for your insightful suggestion. We fully agree that single-cell RNA sequencing (scRNA-seq) offers unique advantages in resolving macrophage polarization states, providing high-resolution, cell type–specific information critical for mechanistic studies. Following your recommendation, we have incorporated analyses based on publicly available scRNA-seq datasets in the revised manuscript, with detailed descriptions provided in the Materials and Methods and Results sections. These analyses further validate, at the single-cell level, the expression patterns of the hub genes across different macrophage polarization states, offering more direct evidence of their regulatory roles in the immune microenvironment of spinal cord injury. This addition substantially enhances the depth and persuasiveness of the manuscript. We greatly appreciate your insightful guidance, which has been instrumental in improving the quality of our study.

- To firmly establish the involvement of the identified hub genes in macrophages, the use of co-localization techniques (e.g., double immunofluorescence or in situ hybridization) is recommended. This would strengthen the claim that these genes are functionally active in macrophage populations post-SCI.

Re: We sincerely thank you for this highly valuable suggestion. We fully agree that applying co-localization techniques, such as double immunofluorescence, to directly validate the expression of target genes in macrophages in situ is highly meaningful. In the current study, bioinformatic predictions (including single-cell analyses) and in vivo RT-qPCR validations have allowed us to preliminarily establish the association of the hub genes with macrophage polarization following SCI. The co-localization approaches you suggested, such as double immunofluorescence staining, constitute a crucial part of our future research plans. This experiment will greatly enhance the rigor and depth of our subsequent work. We sincerely appreciate your guidance in identifying this key direction for further validation.

- Although the study briefly discusses the biological roles of *Soat1*, *Comt*, and *Myo1f*, a more in-depth analysis of their functions in neuroinflammation and SCI — supported by relevant literature — would be valuable.

Re: We sincerely thank you for this insightful suggestion. We fully agree that providing a more in-depth interpretation of the functions of the hub genes in neuroinflammation and spinal cord injury is essential for enhancing the biological insights of the study. In response to your valuable comments, we have substantially revised and expanded the Discussion section, incorporating supporting literature to deepen the functional analysis of the hub genes and to provide new perspectives for future mechanistic investigations.

- Literatures are taken from old papers where enhanced version of clustering techniques are available. So, add some more recent literatures and compare the work with proposed one.

Re: We sincerely thank you for reviewing our manuscript and providing valuable comments. First, we would like to clarify that the core analyses in the initial version of the manuscript did not involve clustering methods. Nevertheless, we fully understand your suggestion to incorporate current methodological advances. In response, we have included a single-cell RNA sequencing (scRNA-seq) analysis module in the revised manuscript, in which UMAP was used for dimensionality reduction and visualization, and relevant recent literature has been cited. We greatly appreciate your constructive recommendation.

- The section about the future study should be mentioned in the last paragraph of the Conclusion section.

Re: We sincerely thank you for your valuable suggestion regarding the manuscript structure. In response, we have adjusted the structure of the manuscript and consolidated the discussion of future research directions into a distinct, focused paragraph that clearly summarizes the avenues for further exploration based on our findings. We greatly appreciate your guidance in helping us improve the logical flow of the manuscript.

- Please expand on how the findings can be practically integrated into clinical or public health strategies?

Re: Thank you very much for this highly valuable suggestion, which prompted us to further reflect on and articulate the potential translational implications of our findings. Following your advice, we have added a new section in the Discussion that outlines the possible pathways for applying our results, including their clinical translation potential and relevance for public health strategies. Your guidance has greatly helped us broaden the scope of our study.

- The quality of the figures should be improved.

Re: Dear Reviewer, thank you very much for your valuable suggestion regarding the quality of the figures. We fully agree that high-quality figures are essential for presenting research results clearly and accurately. In response, we have carefully reviewed and improved all figures in the manuscript, ensuring that their resolution is no less than 300 dpi to meet journal requirements and remain clear even when enlarged. We believe these improvements will help convey our findings more effectively. Thank you again for helping us enhance the presentation quality of our manuscript.

6. PLOS authors have the option to publish the peer review history of their article (what does this mean?). If published, this will include your full peer review and any attached files.

If you choose “no”, your identity will remain anonymous but your review may still be made public.

Do you want your identity to be public for this peer review? For information about this choice, including consent withdrawal, please see our Privacy Policy.

Reviewer #1: Yes: Abubakari Sumaila Salpawuni

Reviewer #2: No

We confirm that we have followed the journal’s requirements and used the Precheck Analysis and Conversion Engine (PACE) tool to review and optimize all figure files, ensuring that they fully comply with PLOS ONE’s publication standards.
