# Supplementary material for: Screening macrophage polarization genes in spinal cord injury as therapeutic targets
Source: PLoS One. 2026 May 4;21(5):e0347599. doi: 10.1371/journal.pone.0347599 (PMC13138653; doi:10.1371/journal.pone.0347599)
Supplement: Attachment — Submitted filename: Response_to_Reviewers_auresp_2.docx [file pone.0347599.s016.docx]

**Point-by-point response to the editor and reviewer comments**

**Dear Editor and Reviewers,**

Thank you very much for your continued evaluation of our manuscript and for your insightful comments and valuable suggestions. We sincerely appreciate your time and effort throughout the review process.

We have carefully revised the manuscript again in response to all the comments raised in this round of review. A detailed, point-by-point response to each comment is provided below. All changes have been clearly indicated in the revised manuscript. We have made every effort to address all remaining concerns and further improve the clarity, rigor, and completeness of the study.

We hope that the revised manuscript now meets the requirements for publication in PLOS ONE. Please do not hesitate to contact us if further clarification is needed.

Journal Requirements:

1. If the reviewer comments include a recommendation to cite specific previously published works, please review and evaluate these publications to determine whether they are relevant and should be cited. There is no requirement to cite these works unless the editor has indicated otherwise.

Re: N/A。

2. Please review your reference list to ensure that it is complete and correct. If you have cited papers that have been retracted, please include the rationale for doing so in the manuscript text, or remove these references and replace them with relevant current references. Any changes to the reference list should be mentioned in the re-buttal letter that accompanies your revised manuscript. If you need to cite a retracted article, indicate the article’s retracted status in the References list and also include a citation and full reference for the retraction notice.

Re: Thank you for your suggestion. We have carefully reviewed and standardized the reference list to ensure its accuracy and completeness. We identified that Reference 35 in the previous version, Retracted Article: Strontium-doped gelatin scaffolds promote M2 macrophage switch and angiogenesis through modulating the polarization of neutrophils, had been retracted.

In accordance with the journal’s requirements, we removed this reference and replaced it with a relevant and valid article of similar scientific significance: Macrophage polarization-related gene SOAT1 is involved in inflammatory response and functional recovery after spinal cord injury. The manuscript has been updated accordingly.

Review Comments to the Author
Please use the space provided to explain your answers to the questions above. You may also include additional comments for the author, including concerns about dual publication, research ethics, or publication ethics. (Please upload your review as an attachment if it exceeds 20,000 characters)

Reviewer #1: This revised manuscript has been significantly improved. In the original version, my concerns largely revolved around issues of preprocessing (e.g., small sample size), lack of detailed discussion of the SCI hub genes screened, language clarity, and failure to acknowledge the limitations of the present study. All these issues have largely been addressed now.

Re：Thank you very much for your careful evaluation and valuable comments. We are pleased that the current revision has largely addressed the concerns you previously raised, including data preprocessing, in-depth discussion of SCI-related hub genes, language clarity, and the acknowledgment of study limitations. We sincerely appreciate your professional guidance.

Minor suggestion:

Since the GSE45006 and GSE45550 datasets were used to screen the DEGs (using the `limma` package) for the downstream analyses, the findings in the study can further be strengthened using the DExMA package or the `Robust Rank Aggregation` (RRA) package for meta-analysis of the GSE45006 and GSE45550 datasets. Using these two packages, can the approach used in DOI: https://doi.org/10.3390/diagnostics15141770 be applicable to your study? How are your findings strengthened?

Re：We sincerely thank you for your valuable suggestion. In response, we have performed additional analyses, and the results have been incorporated into the revised manuscript (without track changes) (lines 151–157, 350-353). The corresponding results have also been provided in the Supplementary Materials for your review.

Specifically, after identifying the hub genes, we applied the same significance thresholds to screen differentially expressed genes (DEGs) from the GSE45006 and GSE45550 datasets. We then used the RobustRankAggreg (RRA) package to integrate the ranked DEG lists from the two datasets. The aggregateRanks function was employed to calculate integrated scores and generate a cross-dataset ranked gene list. Based on this, we evaluated the ranking positions of the three hub genes (Soat1, Myo1f, and Comt) within the integrated list.

The rankings and corresponding RRA scores of the three hub genes are as follows: Soat1 ranked 1,336 (top 8.84%, Score = 0.0936), Myo1f ranked 1,605 (top 10.62%, Score = 0.1139), and Comt ranked 2,972 (top 19.66%, Score = 0.2258). Although these genes were not among the top 20 or top 100 in the integrated list, they were consistently retained in the RRA results and ranked within the upper-middle range across all genes.

Since links to web tools are susceptible to changes in the future, the authors are strongly advised to include the date accessed for the web links in their study. As an example, the authors state“Queries in the Drug-Gene Interaction Database (DGIdb; https://dgidb.genome.wustl.edu/) relied on individual hub genes …”

It should be

“Queries in the Drug-Gene Interaction Database (DGIdb; https://dgidb.genome.wustl.edu/ [Accessed: June 23, 2025]) relied on individual hub genes …” Notice that in the second case, we indicate the web tool was accessed on June 23, 2025.

Re：Thank you very much for your valuable suggestion. In response, we have added the specific access dates for all web-based tools used in this study and standardized the citation format of online resources. These revisions have been incorporated into the manuscript, improving the rigor and clarity of our presentation.

Reviewer #3: I reviewed the revised manuscript which respond to the comments of previous reviewers who mostly focused on the data gathering and bioinformatic analysis. In summary this manuscript tried to identify the genes involved in macrophages polarization after SCI by recruiting data bases and doing bioinformatic analyses. They find three genes in their research and then bring them to the bench in order to evaluate their mRNA expression. I have some comments to more polish the animal study.
-Duration of receiving pain killers and antibiotic should be stated.

-clear group description and number of animals in each group required.

Re：We sincerely thank you for your valuable suggestions, which have greatly contributed to improving the methodological clarity and rigor of the animal experiments in this study. In response to your comments regarding the need for a clear description of experimental groups and the number of animals in each group, as well as the duration of analgesic and antibiotic administration, we have carefully revised and supplemented the Methods section as follows:

At the beginning of the animal model description, we have clearly defined the experimental groups as one sham-operated group and three SCI groups corresponding to 3, 7, and 14 days post-injury. We have explicitly stated the initial number of animals in each group (8 rats per SCI group and 7 rats in the sham group), as well as the final number of animals included in the analyses (7 rats per group across all four groups).

In addition, in the postoperative care section, all analgesics and antibiotics were administered daily after surgery and continued for up to seven consecutive days, or until the day of sacrifice for animals in the 3-day group. Treatment was discontinued once rats regained spontaneous urination and showed no obvious signs of pain or infection.

All relevant revisions have been clearly indicated in the revised manuscript (see Section 2.12, “Spinal cord injury rat model”).

- use one format of genes names. International abbreviation usage is required such as Comt change to COMT, Myo1f to MYO1F and so on.

Re：Thank you very much for your valuable suggestion regarding gene nomenclature. According to the official rat gene naming conventions (RGD), rat gene symbols are formatted with only the first letter capitalized (e.g., Comt, Myo1f, Soat1). To maintain standard academic conventions for rat model studies, we have consistently applied the standardized rat gene nomenclature throughout the manuscript, ensuring uniformity and correctness.

The inconsistencies in gene name capitalization that you noted mainly occurred in the enrichment analysis, IPA analysis, and molecular regulation-related sections. These discrepancies arose because the study involved homologous gene conversion between rat and human, where some genes were presented according to their human gene annotation formats, leading to differences in capitalization.

In summary, we sincerely thank you for your valuable comment. Your careful review and professional suggestions have greatly contributed to improving the quality of our manuscript, and we have carefully revised the text accordingly.

- fig-9- To me the level of expression of desired gene in M1 and M2 macrophages does not have significant difference.

Re：Thank you very much for your careful evaluation of the results presented in Figure 9. We have supplemented the analysis with detailed statistical testing. Specifically, the Wilcoxon rank-sum test was applied to assess differential expression of the three genes. The raw P values for Soat1, Comt, and Myo1f were 1.09 × 10⁻^22^, 1.95 × 10⁻⁹⁹, and 1.61 × 10⁻^181^, respectively. After Bonferroni correction for multiple comparisons, all adjusted P values remained far below 0.0001, indicating extremely strong statistical significance.

The corresponding log2 fold changes were 1.722, 1.555, and 3.920, respectively, all exceeding a two-fold change, thus demonstrating clear biological relevance. In terms of cellular expression patterns, all three genes showed high expression in M1 macrophages and were nearly absent in M2 macrophages, with no overlap in the interquartile ranges between the two groups, indicating a distinct distribution difference.

We have also optimized the figure by annotating adjusted P values and log2FC, and by adding scatter plots to display single-cell expression distributions, making the results more intuitive.

Taken together, these results demonstrate that the three hub genes exhibit highly significant and biologically meaningful differences in expression between M1 and M2 macrophages. We sincerely appreciate your valuable suggestion.

- for functionality of a gene usually we check the end product of a gene which is protein level. I didn't see any western blot or ELISA study in this work.

Re：Thank you very much for your valuable suggestion. We fully agree that validation at the protein level is important for elucidating gene function. In this study, based on a rat spinal cord injury model, we systematically assessed the expression changes of key genes at three consecutive time points (3, 7, and 14 days post-injury) using RT-qPCR. The results demonstrated highly consistent, robust, and time-dependent expression patterns across these time points, providing strong transcriptional evidence supporting the reliability and dynamic regulation of these genes during the pathological progression of spinal cord injury.

We acknowledge that protein-level validation, such as Western blot or ELISA, was not performed in the present study. In future work, we will build upon these findings to further investigate the expression and functional roles of these genes at the protein level. We sincerely appreciate your professional and insightful comments.

- I need to see a clear suggestion for future studies in conclusion section showing us based on this study who can we reach to maximum protection in SCI by manipulating these genes.

Re：Thank you very much for your important suggestion. In response, we have revised the Conclusion section to include future research directions based on our findings. Specifically, we propose that modulation of the key genes identified in this study may further elucidate their neuroprotective roles in spinal cord injury, thereby providing potential therapeutic targets and experimental evidence for maximizing neuroprotection after SCI.

In addition, future studies may incorporate strategies such as gene overexpression, knockdown, or pharmacological targeting to investigate the synergistic regulatory effects of these genes, with the aim of optimizing approaches for clinical translation and functional recovery.

We sincerely appreciate your valuable comments, which have greatly improved the completeness and significance of our manuscript.

- Taken together, this study brings new hypothesis which needs to be evaluated by further animal and clinical trial studies.

Re：Thank you very much for your valuable comments. We fully agree with your perspective. In the present study, the expression of the hub genes was only preliminarily validated using RT-qPCR, and the hypotheses generated still require further verification through more in-depth experimental and clinical studies.

In the revised manuscript, we have highlighted these limitations in the Discussion section. Future work will focus on mechanistic validation in animal models as well as clinical translation, in order to systematically evaluate the reliability and applicability of this hypothesis. We sincerely appreciate your insightful suggestions, which have provided important direction for our future research and have greatly improved the rigor and completeness of this manuscript.
